# Modeling early changes associated with cartilage trauma using human-cell-laden hydrogel cartilage models

**DOI:** 10.1186/s13287-022-03022-8

**Published:** 2022-08-04

**Authors:** Chunrong He, Karen L. Clark, Jian Tan, Hecheng Zhou, Rocky S. Tuan, Hang Lin, Song Wu, Peter G. Alexander

**Affiliations:** 1 Department of Orthopaedic Surgery, Center for Cellular and Molecular Engineering, University of Pittsburgh School of Medicine, 450 Technology Drive, Room 213, Pittsburgh, PA 15219 USA; 2 The Third Hospital of Xiangya, Central South University, Changsha, 410013 Hunan China; 3 Institute for Tissue Engineering and Regenerative Medicine, The Chinese University of Hong Kong, Shatin, Hong Kong SAR China

**Keywords:** Human mesenchymal stem cells, 3D cartilage models, Methacrylated gelatin chondrogenesis, Traumatic impact loading, Post-traumatic osteoarthritis (PTOA)

## Abstract

**Background:**

Traumatic impacts to the articular joint surface are known to lead to cartilage degeneration, as in post-traumatic osteoarthritis (PTOA). Limited progress in the development of disease-modifying OA drugs (DMOADs) may be due to insufficient mechanistic understanding of human disease onset/progression and insufficient in vitro models for disease and therapeutic modeling. In this study, biomimetic hydrogels laden with adult human mesenchymal stromal cells (MSC) are used to examine the effects of traumatic impacts as a model of PTOA. We hypothesize that MSC-based, engineered cartilage models will respond to traumatic impacts in a manner congruent with early PTOA pathogenesis observed in animal models.

**Methods:**

Engineered cartilage constructs were fabricated by encapsulating adult human bone marrow-derived mesenchymal stem cells in a photocross-linkable, biomimetic hydrogel of 15% methacrylated gelatin and promoting chondrogenic differentiation for 28 days in a defined medium and TGF-β3. Constructs were subjected to traumatic impacts with different strains or 10 ng/ml IL-1β, as a common comparative method of modeling OA. Cell viability and metabolism, elastic modulus, gene expression, matrix protein production and activation of catabolic enzymes were assessed.

**Results:**

Cell viability staining showed that traumatic impacts of 30% strain caused an appropriate level of cell death in engineered cartilage constructs. Gene expression and histo/immunohistochemical analyses revealed an acute decrease in anabolic activities, such as *COL2* and *ACAN* expression, and a rapid increase in catabolic enzyme expression, e.g., *MMP13*, and inflammatory modulators, e.g., *COX2*. Safranin O staining and GAG assays together revealed a transient decrease in matrix production 24 h after trauma that recovered within 7 days. The decrease in elastic modulus of engineered cartilage constructs was coincident with GAG loss and mediated by the encapsulated cells. The acute and transient changes observed after traumatic impacts contrasted with progressive changes observed using continual IL-1β treatment.

**Conclusions:**

Traumatic impacts delivered to engineered cartilage constructs induced PTOA-like changes in the encapsulated cells. While IL-1b may be appropriate in modeling OA pathogenesis, the results of this study indicate it may not be appropriate in understanding the etiology of PTOA. The development of a more physiological in vitro PTOA model may contribute to the more rapid development of DMOADs.

**Supplementary Information:**

The online version contains supplementary material available at 10.1186/s13287-022-03022-8.

## Introduction

Osteoarthritis (OA) is the most prevalent joint disease, affecting over 500 million individuals globally [1, 2]. The majority of people suffering OA are over the age of 65; however, joint trauma is a major cause of OA in young patients, where studies link injury, for example in the knee, in young adults to a fourfold to sevenfold increased risk for knee OA by middle age [3–5]. Despite the high burden of OA in society and research resources expended in understanding the etiology and pathophysiology of the disease, there are no FDA-approved disease-modifying OA drugs (DMOADs) to treat or prevent the disease [6].

Limited progress in DMOAD development may be due to insufficient mechanistic understanding of human disease onset and progression stemming from insufficient in vitro models for disease and therapeutic modeling [7–9]. Of note, congruency between clinical outcomes and the data derived from in vitro and preclinical models for DMOADs are especially low [10–12], necessitating the development of affordable, in vitro human cell-based models that allow for direct mechanistic analysis of target cell populations [10, 13].

In developing in vitro models of cartilage, hydrogels have proven very effective in providing a viscoelastic environment suitable for promoting and supporting the cartilage phenotype [16]. Many studies have demonstrated the ability of human bone marrow-derived mesenchymal stem cells (MSCs) to undergo chondrogenic differentiation and elaborate function matrix when encapsulated within various hydrogels in vitro and in vivo [17]. MSC- and chondrocyte-based hydrogel models of cartilage have been important in establishing the importance of mechanical stimulation in inducing and increasing matrix elaboration[18] and the ability of chronic overloading to induce catabolic activities similar to that observed in vivo [19], supporting the physiological relevance of these models.

The utility of in vitro engineered cartilage models for the study of pathogenic mechanisms and therapeutic interventions following acute joint trauma has not been as successful. Acute trauma, defined by acute high load and strain, is difficult to replicate with soft hydrogels. Though a characteristic of OA, the use of inflammatory cytokines such as IL-1β and TNF-α to induce a chondrocyte disease state is more appropriate to the study of rheumatoid arthritis (RA), not OA [20–24]. And, while mechanical disruption using high strain dynamic loading (up to 25% at 1 Hz) has been shown to induce OA-like cell responses in MSC-laden hydrogel [19], these models do not generate the etiology of PTOA because they do not use acute loads to initiate the onset of OA.

The goal of this study is to develop a model of PTOA using acute injury onset and engineered cartilage models for deployment in a mesoscale microphysiological microJoint chip [14, 15]. As a first step, we hypothesized that a single traumatic impact to human cell-based engineered cartilage models induces appropriate, physiological markers of early PTOA similar to those observed in in vivo and in vitro model, including changes in cell viability, changes in cell gene expression and matrix elaboration.

## Methods

### Materials and reagents

All reagents were purchased from Sigma-Aldrich unless otherwise stated. Methacrylated gelatin (GelMA) was obtained through the reaction between gelatin and methacrylic anhydride (MA) as previously described [25]. The photoinitiator lithium phenyl-2,4,6-trimethylbenzoylphosphinate (LAP) was synthesized as previously described by Fairbanks et al. [26]. The impactor employed was used as previously described [27].

### Culture media

hBM-MSC growth medium (GM): Dulbecco's Modified Eagle Medium (DMEM) supplemented with 10% fetal bovine serum (FBS) and 2% penicillin/streptomycin/fungizone; hMSC chondrogenic medium (CM): GM without FBS, supplemented with 10 ng/mL TGF-β3, 1% insulin-transferrin-selenium, 50 μM L-ascorbic acid-2 phosphate, 10 nM dexamethasone and 23 μM proline; chondrogenic basic medium: Dulbecco's Modified Eagle Medium (DMEM) supplemented with 1% insulin-transferrin-selenium, 23 μM proline and 2% penicillin/streptomycin/fungizone.

### Cell culture

hBM-MSCs were obtained, with IRB approval of the University of Pittsburgh, from femoral heads of patients who underwent total joint arthroplasty, according to a previously described procedure [52]. hBM-MSCs were cultured as monolayer in GM at 37 °C and 5% CO_2_. GM was changed every 2‒3 days until ~ 80‒90% confluency. Aliquots of cells were collected before plating (p4) for CFU (Additional file 1: Fig. S1); tri-lineage differentiation (Additional file 2: Fig. S2); and surface antigen profile (Additional file 3: Fig. S3). All experiments were performed in triplicates, using cells pooled from 8 different donors (mean age = 54.5 yo, age range = 38–76 yo).

### Engineered cartilage construct preparation

After trypsinization, passage 4 (p4) hBM-MSCs, usually 9 T175 (Falcon flasks of 80% confluent cells) containing 24–27 million cells, were washed twice in Ca^2+^/Mg^2+^-free HBSS. After careful removal of the supernatant above the final pellet, the cells were resuspended in a volume of 15%(w/v) liquid GelMA/LAP to produce a concentration of 20 million cells per ml. Hydrogel constructs, 2 mm(h) × 5 mm (*∅*), were prepared by aliquoting 40 μL cell/hydrogel suspensions into silicone molds, comprising a 5 × 5 array of 5-mm-diameter holes produced by biopsy punch in a 4 cm × 4 cm square piece of 2-mm-thick silicone, that were placed in a sterile 100-mm cell culture dish. After deposition of the liquid cell suspension into the mold, a coverslip was placed on top and the cell/hydrogel mixture was exposed to UV light from a dental lamp (wavelength 430–490, power 1400 mw/cm^2^) for 120 s [17, 54]. After polymerization, the constructs were removed from the mold, trimmed of any excess hydrogel with a scalpel, placed into chondrogenic medium and allowed to differentiation for 28 days (Additional file 4: Fig. S4).

### Traumatic impact delivery

**Fig. 1:**
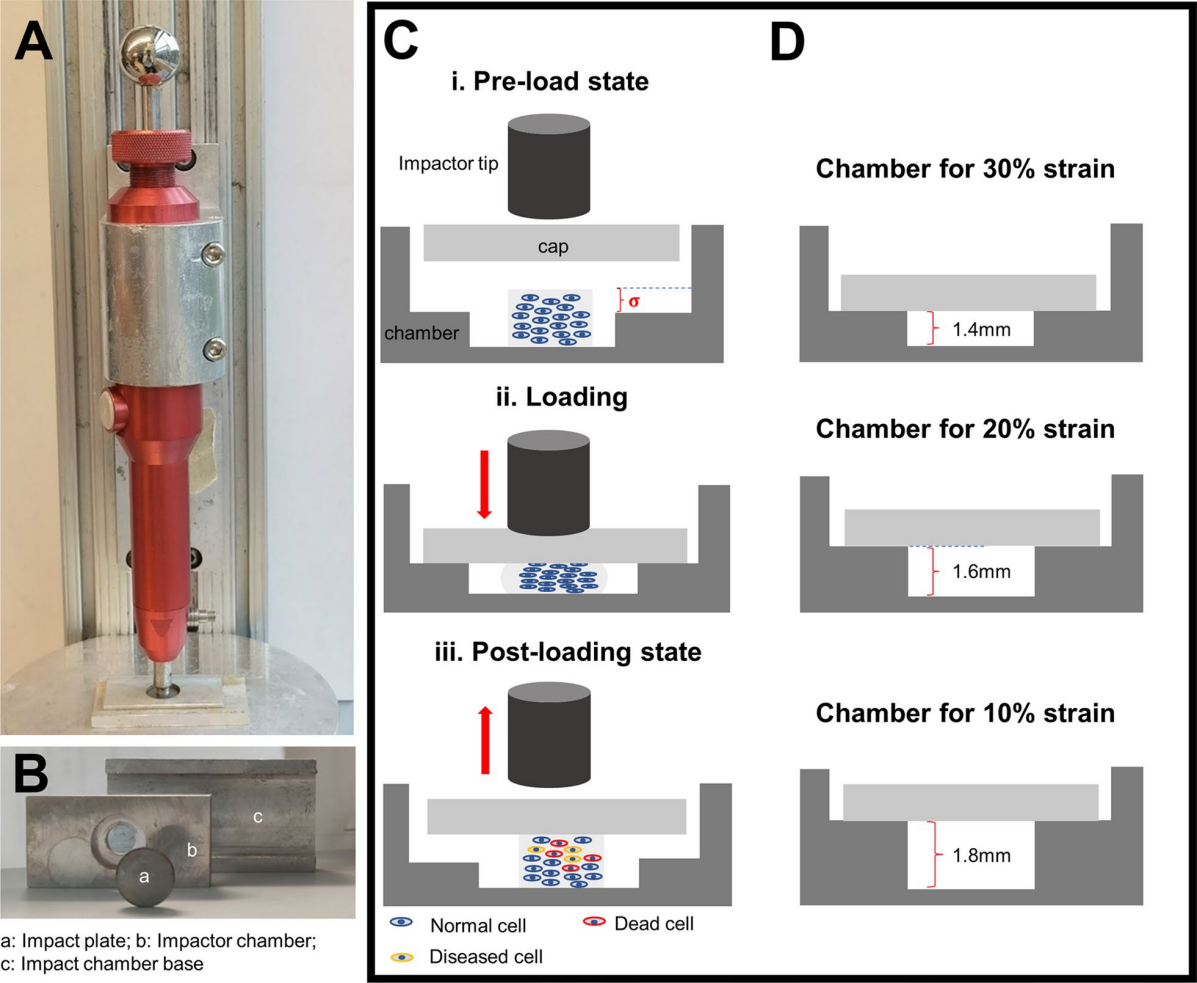
Mechanism to deliver traumatic impacts to hydrogel-based MSC-derived engineered cartilage constructs. **A** Spring-loaded impact device in vertical alignment. **B** Chambers fabricated to restrict compression of hydrogel to a specific strain protect hydrogel from being crushed while subjected to a traumatic load. **B** Mechanism of how impact loadings of specific strain were delivered. **C** Schematic depicting means of delivering traumatic impact loads of a specific strain to a hydrogel. **D** Schematic depicting different chambers for the delivery of impacts with specific strains

An impactor system (Fig. 1) previously used to deliver single traumatic impacts to articular cartilage in vivo and ex vivo was utilized for impact loading [24, 27]. The essential characteristic used in this study is the ability of the device to deliver a single impact at a very high strain rate (up to 30%). As the hydrogels used here are not able to withstand high-energy impact loading, a special chamber system (Fig. 1B–D) was designed to protect hydrogel constructs from being crushed during unconfined impact loading. Engineered cartilage constructs were subjected to high velocity (< 1 ms time to peak). The percent strain of impacts was regulated by restricting the movement of the impact plate. This was accomplished by fabricating chambers with ledges to stop the progression of the impact plate at specific heights corresponding to 10, 20 and 30% strain of a 2-mm-tall construct (at heights of 1.8, 1.6 and 1.4 mm from the bottom of the loading chamber). Traumatic impacts were delivered to hydrogel constructs 28 days after differentiation in chondrogenic medium. IL-1β stimulation was used as a positive control and comparative treatment. Outcomes were analyzed at 1, 3 and 7 days after impact loading or IL-1β stimulation. Engineered cartilage models were put into chondrogenic basic medium after stimulation until collected for analysis. All processes related to delivering a traumatic impact were conducted using a-septic technique in a sterile biosafety cabinet.

### Live–dead staining

Twenty-four hours after trauma or IL-1β treatment, engineered cartilage constructs were bisected transversely and stained with Calcein AM (Invitrogen, Cat# L3224A) and ethidium homodimer (Invitrogen, Cat# L3224B) for 45 min in a 37 °C incubator. Images of the cut, transverse plane through the center of the construct were taken using a confocal microscope (Olympus FluoView FV1200) set for red (527_ex_ nm/624_em_ nm) or green (488_ex_ nm/520_em_ nm) detection. 40 × and 100 × magnification were used to take pictures. Ten stacks for each construct were taken with a z-stack density of 10 um. Fiji ImageJ software was used to process images and quantify live and dead cells.

### Cell Counting Kit 8(WST-8) assay

Cell metabolic activity was quantitated. Cell Counting Kit 8 (WST-8 / CCK8) (Abcam, ab228554) was used to test cell viability and metabolic activity of cells within the engineered cartilage constructs. The constructs were collected and submerged in WST-8 solution and incubated in a 37 °C incubator for 2 h, under agitation. The absorbance (460 nm) of the WST-8 solution collected after incubation was measured by a microplate reader (BioTek).

### Elastic modulus

A MATE (mechano-active tissue engineering) system was used to measure elastic modulus of engineered cartilage constructs and hydrogels without cell seeded [53]. A special 6-well plate for the MATE system was used to put the constructs on the MATE system, and analysis mode was used to measure elastic modulus of the constructs. A safe strain of 30% was set to protect the constructs from being crushed. The constructs were loaded to 30% strain in the first second of the test (Fig. 4A) and then allowed rest under that deformation for an additional 10 s. The force–strain curve of each sample was generated from these data (Fig. 4B), and the elastic modulus was calculated using the MATE software according to these force–strain curves and sample size (2 mm (h) × 5 mm (*∅*)).

### Gene expression analysis

Total RNA was extracted from the cells within engineered cartilage constructs by grinding the constructs with motorized pestles (Fisherbrand, cat #12141363 of the motor and pestle) within 1.5-ml Eppendorf tubes, fully mixing the suspension in TRIzol reagent (Ambion, cat#15596018) and isolating purified nucleic acid according to the manufacturer’s instruction. An RNeasy Plus Universal Mini Kit (QIAGEN, cat#74136) was used to purify the RNA. RNA concentration of the final products was measured using a NanoDrop 2000C (Thermo Scientific). Purified RNA extracts were reverse-transcribed into cDNA using SuperScript IV Reverse Transcriptase (Invitrogen, cat #18090050). The cDNA was used for subsequent qPCR in a QuantStudio 3 real-time PCR system (Applied Biosystems) and SYBR Green Reaction Mix (Applied Biosystems, cat#A25742) with a maximum cycle of 40. The expression of COL2A1, ACAN, SOX9, MMP13, ADAM-TS4, ADAM-TS5, INOS and PTGS2 was detected through qRT-PCR (Table 1).

**Table 1:** Primer sequences for qRT-PCR analysis of gene expression

	Forward 5′–3′	Reverse 5′–3′
*RPL13A*	GCCATCGTGGCTAAACAGGTA	GTTGGTGTTCATCCGCTTGC
*COL2A1*	TGGACGATCAGGCGAAACC	GCTGCGGATGCTCTCAATCT
*ACAN*	ACTCTGGGTTTTCGTGACTCT	ACACTCAGCGAGTTGTCATGG
*SOX9*	AGCGAACGCACATCAAGAC	CTGTAGGCGATCTGTTGGGG
*MMP13*	ACTGAGAGGCTCCGAGAAATG	GAACCCCGCATCTTGGCTT
*ADAMTS4*	GAGGAGGAGATCGTGTTTCCA	CCAGCTCTAGTAGCAGCGTC
*ADAMTS5*	GAACATCGACCAACTCTACTCCG	CAATGCCCACCGAACCATCT
*INOS*	TTCAGTATCACAACCTCAGCAAG	TGGACCTGCAAGTTAAAATCCC
*PTGS2*	CTGGCGCTCAGCCATACAG	CGCACTTATACTGGTCAAATCCC

### Histology and immunohistochemistry

Samples were fixed in 10% neutral buffered formalin for 24 h and thereafter went through an ethanol dehydration series, xylene exchange and paraffin embedding following standard procedures, and sectioned at 6 µm thickness. For histology, sections were rehydrated and stained with hematoxylin (Harris cat #HHS16, Sigma-Aldrich) and eosin (alcoholic, Sigma-Aldrich, cat#HT110132) or Safranin O (Sigma 477-73-6) and Fast Green (Sigma 2353-45-9) to study cell morphology and sulfated proteoglycan deposition, respectively. For immunohistochemistry (IHC), sections were pre-incubated for 10 min with 3% H_2_O_2_ in methanol solution to quench endogenous peroxidase activity. Nonspecific binding was then suppressed with 1% horse serum (Vector Labs) in PBS for 45 min. Sections were then incubated overnight at 4 °C with primary antibodies against Collagen II (Invitrogen MA5-12789, 1:100), MMP13 (Invitrogen MA5-14238, 1:25) and ADAM-TS4 (Invitrogen MA5-26715, 1:150) followed by 30-min incubation with biotinylated secondary antibody (Vector Labs). Immunostaining was detected using horseradish peroxidase (HRP)-conjugated streptavidin and Vector® NovaRED™ peroxidase substrate, with hematoxylin (Vector Labs) as counterstain. After staining, both histology and IHC slides were dehydrated, mounted, coverslipped and imaged with microscope (Nikon Eclipse E800).

### Glycosaminoglycan (GAGs) assay

Engineered cartilage samples were washed in PBS and digested in 300 μl papain solution (Sigma-Aldrich P3125, Papain buffered aqueous suspension 50 ×) at 60 °C overnight. After centrifugation at 12,000×*g* for 15 min (room temperature), the supernatant was kept for DNA and GAG quantification. The PicoGreen Assay Kit (Invitrogen cat#P11496) was used to quantify DNA. The glycosaminoglycan (GAG) Detection Assay (Chondrex, cat #6022) was used to quantify the solubilized GAGs. A microplate reader (BioTek) was used to measure absorbance at 656 nm (for GAG), and fluorescence at 520 nm (for DNA) was measured using an HT Synergy microplate reader (BioTek).

### Statistical analysis

Each sample was assayed in triplicate, and the quantitative data were reported as mean ± SD. Statistical analyses were performed using GraphPad Prism 8 (GraphPad Software Inc.). Student's *t* test, and one-way ANOVA/post hoc Tukey test were used to compare two or more independent groups, respectively. Statistical tests were two-tailed, and significance was set at *p* ≤ 0.05, but reported at *p* ≤ 0.05, 0.01, 0.001 or 0.0001, as detailed in each figure.

## Results

### Traumatic impacts induced changes in cell viability

**Fig. 2:**
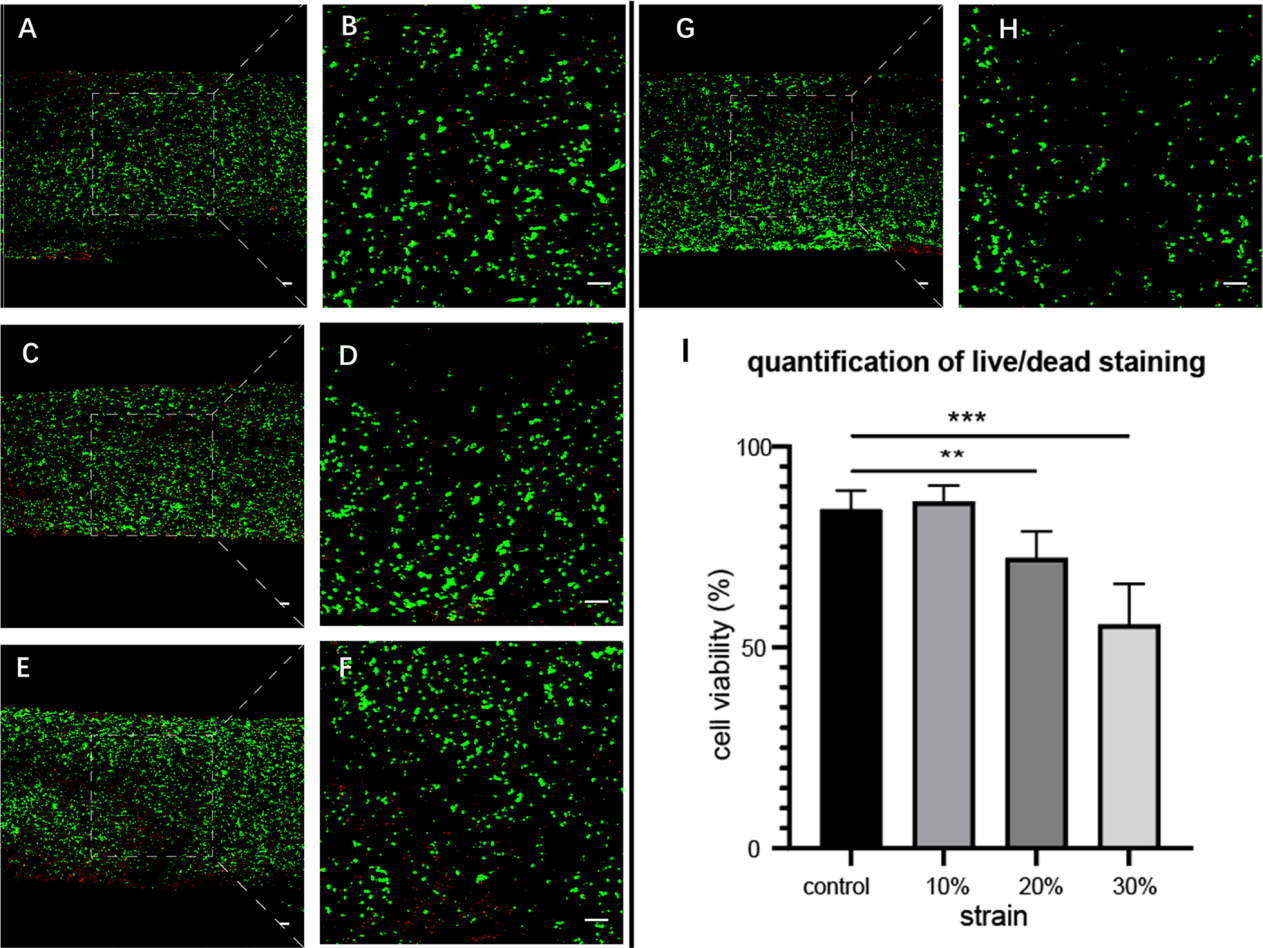
Cell death after traumatic impact Live/Dead™ staining of MCS-derived chondrocyte constructs subject to **A**, **B** 10%, **C**, **D** 20%, **E**, **F** 30% strain of **G**, **H** for impact imaged at **A**, **C**, **E**, **G** 8 × ot **B**, **D**, **F**, **H** 100 × magnification (*n* = 5/group). Green labels live cells and red indicates dead cells. **I** Quantification of cell loss. **p < 0.01, ***p < 0.001. The scale bar in all images = 100 micrometers

**Fig. 3:**
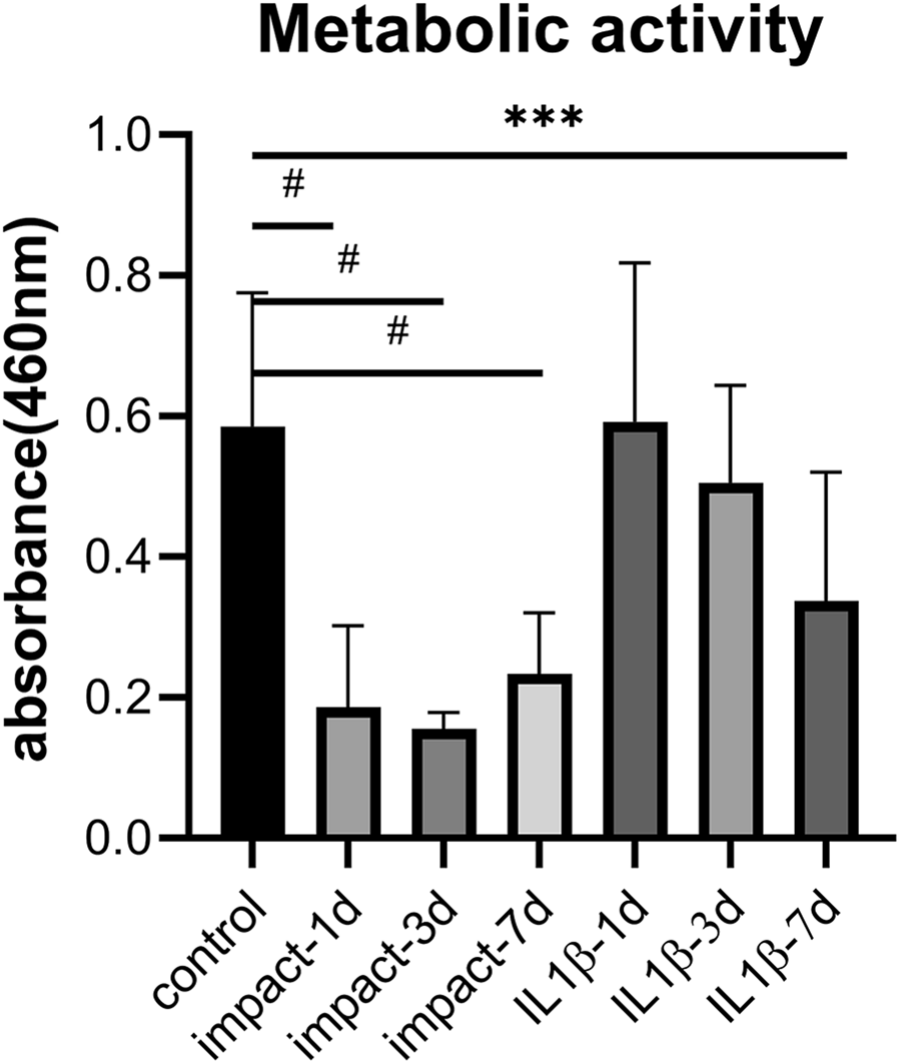
Cell metabolic activity in traumatized engineered cartilage constructs. Cell metabolic activity within engineered cartilage constructs impacted at 30% strain or treated with 10 ng/ml IL-1β was assayed by WST-8/CCK8 assays at 1, 3 and 7 days after treatment. *n* = 20/group, ^#^*p* < 0.0001; ***p < 0.001

We used the Live/Dead™ cell viability test to characterize the effect of traumatic loads with different strains (0, 10, 20 and 30% strain) upon cell viability within the chondrocyte constructs 24 h after loading (Fig. 2). We were interested in inducing a level of cell death greater than 30% previously demonstrated to reliably induce chondrocyte catabolic activities [28]. Microscopic imaging of Live/Dead™ staining imaged in transverse section and assessed for percent live cells revealed that strains of 10% had no significant effect on viability, while strains of 20% and 30% induced 28 ± 6% and 44 ± 9% rates of cell death, respectively. Based on these results, we chose to continue the study with traumatic loads with 30% strain. The CKK8/WST8 assay was then used to more fully characterize changes in cell viability and metabolic activity 1, 3 and 7 days after impact. Exposure to 10 ng/ml IL-1β was used as a comparison for increased catabolic activity. The results (Fig. 3) showed that traumatic loading induced a significant decrease in absorbance at 460 nm (indicating decreased cell viability and metabolic activity) of 68 ± 19% as compared to unimpacted controls after 24 h, with no significant changes observed over the next 6 days. In comparison, IL-1β stimulation induced a steady decrease in absorbance that reached 42% ± 31% by day 7. The absorbance of hydrogels collected 7 days after impact loading increased, which could be regarded as a recovering activity of traumatic engineered cartilage constructs.

### Traumatic impact-induced changes in gene expression

**Fig. 4:**
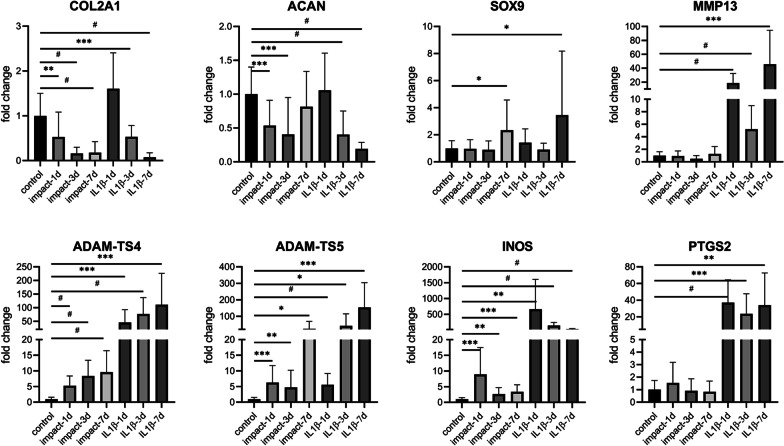
Traumatic impact-induced changes in gene expression. RT-PCR analysis of the expression of genes related to cartilage anabolism (SOX9, COL2A1 and ACAN), cartilage catabolism (MMP13, and ADAM-TS4&5) and cell stress (iNOS and PTGS2) in engineered cartilage constructs subjected to 30% strain versus samples exposed to 10 ng/ml IL-1β at 1, 3 and 7 days of culture. *n* = 20/group. ^#^*p* < 0.0001; ***p < 0.001; **p < 0.01; *p < 0.05

We were now interested in assaying changes in MSC-derived chondrocyte gene expression within the constructs that might be associated with the traumatic impacts. Quantitative RT-PCR (Fig. 4) showed that the expression of *COL2A1* and *ACAN* chondrocyte anabolic genes was downregulated by 3 days after traumatic loading, while that of *SOX9*, a key regulator of cartilage differentiation, was unchanged at day 3. Similarly, IL-1β exposure induced a gradual and increasing reduction in the expression in C*OL2A1* and *ACAN* with an increase in *SOX9* expression detectable on day 7. Whereas *COL2A1* expression remained decreased at day 7, expression of *ACAN* returned to a level statistically equivalent to controls, while *SOX9* expression actually increased by day 7. Similarly, IL-1β exposure induced a gradual and increasing reduction in the expression in *COL2A1* and *ACAN* with an increase in *SOX9* expression detectable on day 7.

The expression of *ADAM-TS4* and *ADAM-TS5*, chondrocyte catabolic genes, was upregulated 1, 3 and 7 days after traumatic impact. Surprisingly, the expression of *MMP13*, a well-established marker of cartilage catabolism and OA, remained low through the 7 days post-impact. The expression of PTGS2, a gene that produces COX2 responsible for prostaglandin E2 production, also remained largely unchanged after traumatic impact. In contrast, *INOS* gene expression was elevated, albeit slightly, after impact at all three time points. In comparison, IL-1β treatment induced much greater increases in the expression of all catabolic genes tested: *MMP13*, *ADAM-TS4*, *ADAM-TS5*, *INOS* and *PTGS2*.

### Traumatic impacts induced changes in matrix elaboration

**Fig. 5:**
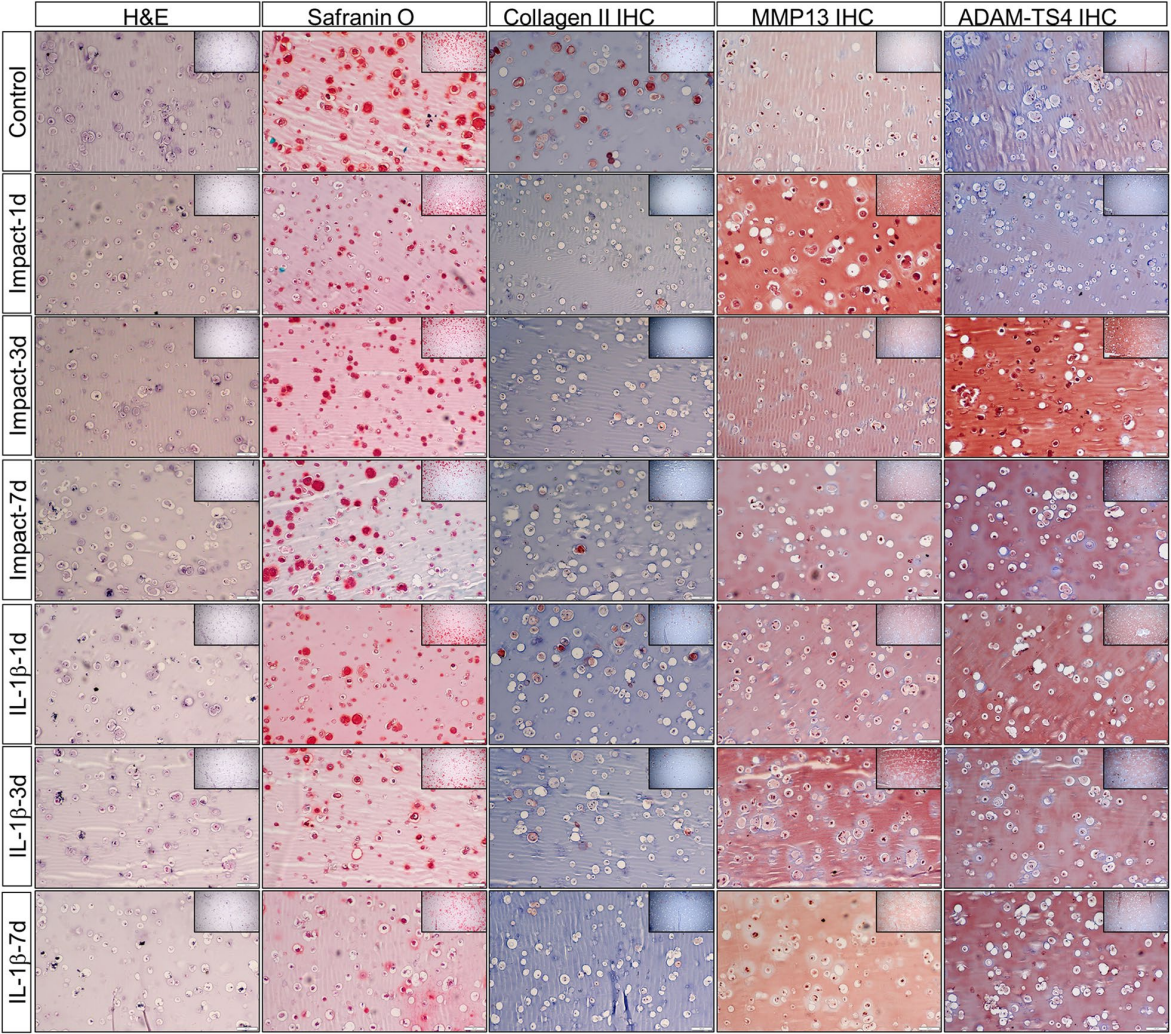
Traumatic impact-induced changes in matrix deposition and catabolic enzyme production. Rows: Engineered cartilage constructs were subjected to 30% strain or exposed to 10 ng/ml IL-1β at 1, 3 and 7 days of culture. Columns: samples were histochemically stained with hematoxylin and eosin or Safranin O/Fast Green or immunohistochemically processed to detect COL2, MMP13 or ADAM-TS4. magnification = 100 × (inset = 40 ×)

We were then interested in confirming some of these results with histochemistry and immunochemistry (Fig. 5). Staining of paraffin-embedded, sectioned samples with hematoxylin and eosin (H&E) indicated both a loss of nuclei and basophilic (blue) matrix with impact with a slight recovery in matrix basophilia by day 7. IL-1β, on the other hand, caused a gradual loss in basophilia that was highest by day 7. Safranin O/Fast Green staining showed that traumatic impacts to engineered cartilage constructs caused a decrease in GAG deposition in the hydrogel constructs evident at 1 day after impact, with an apparent recovery in GAG deposition by day 7, presumably by surviving cells in the constructs. In contrast, IL-1β treatment caused a progressive loss of Safranin O staining during the 7-day culture period (1d, 3d and 7d). COL2A1 immunohistochemistry (IHC) showed that impact loading and IL-1β treatment both caused decreased COL2A1 expression in the hydrogel constructs. Unlike GAG deposition, COL2A1 production/deposition did not recover by the 7-day time point after traumatic loading. IHC for MMP13 in control samples produced light HRP staining in the cells themselves. Traumatic impacts apparently increased MMP13 signal both with the cells and in the matrix, where the intensity of staining decreased to control levels at day 3 and 7. The MMP13 signal in the IL-1β -treated samples was less pronounced but persistent at all experimental time points. ADAM-TS4 staining after traumatic loading increased over time, with the highest signal detected within the cells on day 3, a pattern similar to that observed for IL-1β treatment.

### Traumatic impact-induced reduction in sulfated proteoglycan content and elastic modulus

**Fig. 6:**
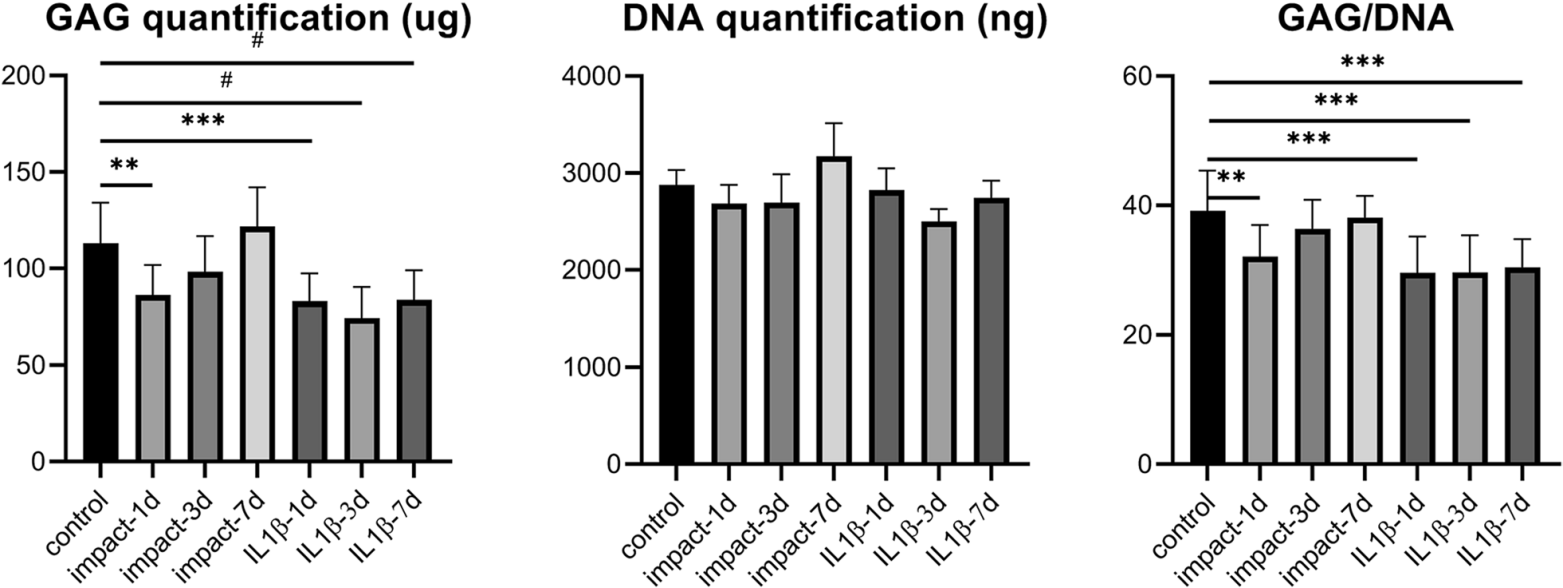
GAG deposition in engineered cartilage constructs after traumatic impact. The DMMB assay was used to quantitate the GAG content in engineered cartilage constructs 1, 3 or 7 days after traumatic impacts or continuous IL-1β treatment. *n* = 12/group. ^#^*p* < 0.0001; ***p < 0.001; **p < 0.01

**Fig. 7:**
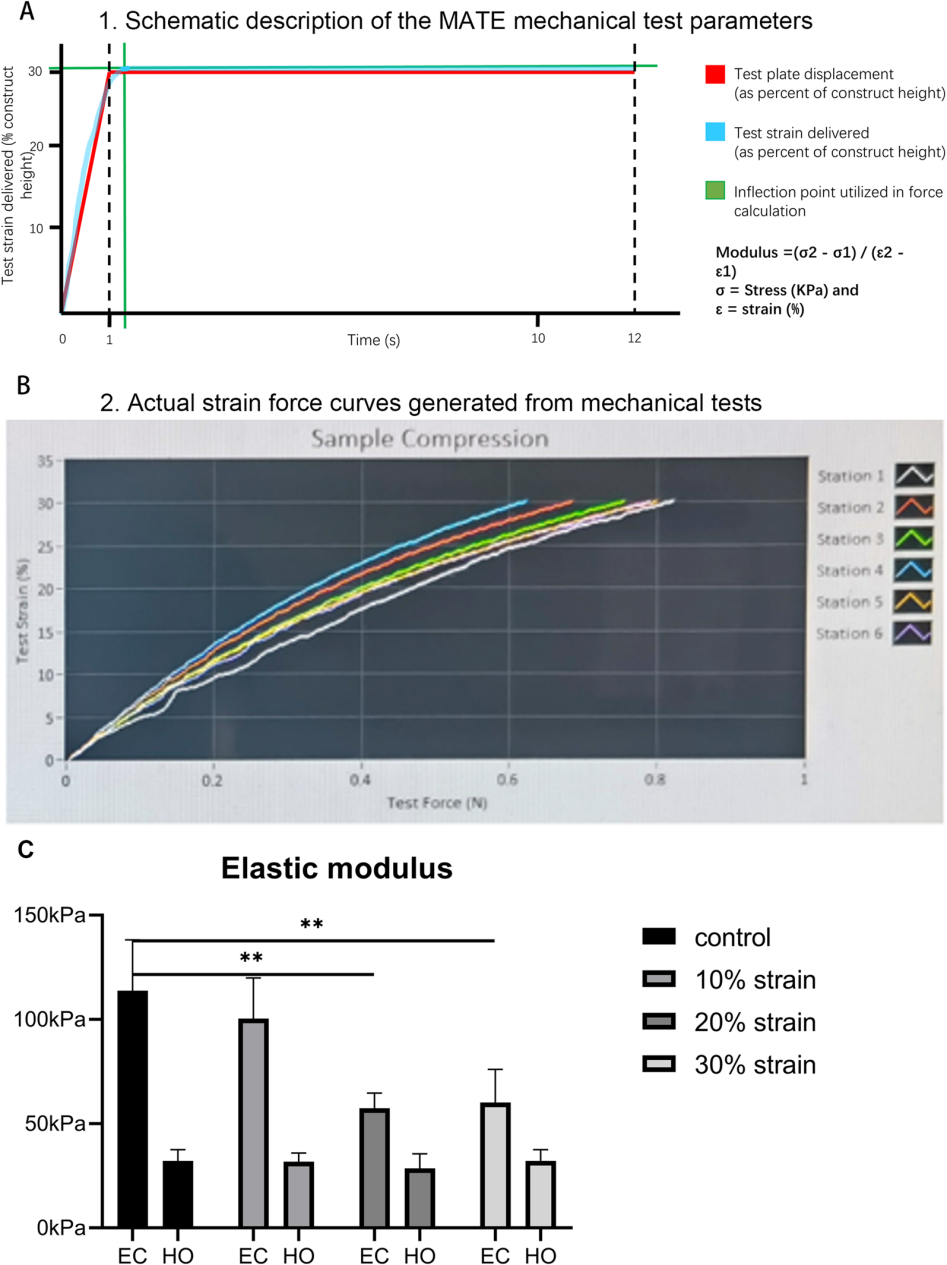
Changes in the mechanical property of engineered cartilage constructs after traumatic loading. Schematic description of the MATE mechanical test parameters (**A**) and MATE strain versus force curves generated from mechanical tests (**B**) The calculated elastic modulus of engineered cartilage constructs and a-cellular hydrogels 24 h after exposure to 0, 10, 20 and 30% strain impacts was determined by uniaxial compression to safe strain (30%) (**C**). EC: engineered cartilage, HO: hydrogel only (without cell seeded). *n* = 5/group. **p < 0.01

We used the glycosaminoglycan detection assay to quantitate the GAG deposition within the constructs (Fig. 6). Our data showed that the traumatic impact caused a rapid decrease in GAG content on day 1 followed by a trending increase in GAG detected on days 3 and 7. In contrast, IL-1β treatment caused a progressive decrease in GAG content in the constructs from day 1 to day 7. Using the MATE system (Fig. 7A,B), we also observed a concomitant decrease in the elastic modulus of the cellular engineered cartilage constructs on day 1 with traumatic impacts of 20 and 30% strain (Fig. 7C). Interestingly, we did not observe a difference between 20 and 30% traumatic loads, suggesting that the viable cell response to load is maximally induced at 20% strain. Although the overall elastic modulus of a-cellular constructs was lower than cell-laden, differentiated chondrocyte constructs, significant decreases after traumatic impact inducing strains up to 30% did not cause a change in this mechanical property of the a-cellular constructs, indicating that the changes in the mechanical properties were due to cell-mediated processes.

## Discussion

In vitro models are essential tools for reductionist, mechanistic research and drug screening. To properly apply information to the clinical conditions, an in vitro system needs to be physiologically relevant. The lack of new FDA-approved for the treatment or prevention of OA, despite our increased understanding of its pathophysiology and application to sophisticated in vivo (rodent) models, suggests that human physiology and tissue interactions need to be accounted for in early benchtop and screening phases of drug development. Recognizing this, the NIH initiated a call for human microphysiological systems (MPS) or tissue chips, in vitro, three-dimensional systems constructed of human cells on bioengineered platforms that recapitulate aspects of in vivo tissue architecture and physiological conditions, to more accurately model tissue physiology for the purpose of (1) disease modeling, (2) drug development, (3) clinical trial design, and (4) chemical and environmental exposures. MPS are increasingly applied to musculoskeletal systems, such as the synovial joint, with impressive results. At least two factors distinguish skeletal models and that of other tissues: (1) the very high matrix-to-cell ratio and (2) the requirement for mechanical stimulation for homeostasis and disease modeling. We have recently reported on the development of an interconnected microchip of joint tissues (microJoint) based on tissue analogs developed with hydrogels and specific stimulants to model inflammatory joint disease, demonstrating physiological responses to therapeutic agents such as nonsteroidal anti-inflammatory drugs (NSAIDs) and experimental peptides [14, 15]. Mechanical loads have been to date difficult to apply to synovial joint MPS, including traumatic loads, the causative event in the development of PTOA and responsible for 10–12% of OA cases. In this study, we are developing an in vitro model of traumatic loads as a first step to characterize a PTOA model in our synovial joint MPS.

A review of studies on chondrocyte construct loading reveals the use of dynamic cyclic compression with physiological loading frequencies with rest periods replicating daily activity and continued exercise over multiple days. In the existing synovial joint MPS [29–36], the mechanical stimulation used has a strain of 0–35% at a frequency of 0.2–1 Hz [51]. These conditions are good for establishing joint tissue homeostasis and possibly modeling chronic overload as a cause of OA under the right (to be determined) conditions. In modeling joint trauma, we adhered to the definitions outlined by Aspden and Burgin: rise times less than 2 ms and one or more of the following traits: loading rates > 100 kN/s, stress rates > 1000 MPa/s or strain rates > 500/s. In explant cultures, the spring-loaded impactor employed in this study at its highest spring compression delivered traumatic impact loads with rise times 1–2 ms in duration, which, at 40 MPa, resulted in stress rates of approximately 20,000/s and calculated strain of 32% at the apex of the hemispherical tip used. In modeling this impact in vitro with engineered tissue, we wanted to maintain the rate of impact and strain without destroying the samples. This is the use of a stop ledge to prevent total destruction of the engineered cartilage constructs.

It has been previously demonstrated that 20 × 10^6^ cells/ml MSCs in a hydrogel can result in construct with sufficient neomatrix that significantly contributes to the properties of the chondrocyte construct. The data presented here indicate that differentiation of the encapsulated MSCs resulted in GAG and COL2 deposition representative of a chondrocyte analog. This is important, as both matrix and water play a role in the forces experienced by chondrocytes in trauma. In traumatic impacts, water motion is largely canceled out and the properties of the matrix and the interactions of the cells with that matrix are dominant. The engineered cartilage constructs used in this study possess an elastic modulus of 30–40kPA and Young’s modulus of 196 ± 21 kPA by 28 days of in vitro differentiation. Upon traumatic loading with 30% strain, we observed a decrease in cell viability compared to that observed with impacts of similar rate and strain [37, 38]. We also observed a loss on elastic modulus which seemed to be dependent on the encapsulated cells, inconsistent with decreased modulus of menisci and articular cartilage of PTOA lapine model [39]. The observed changes in the expression of anabolic genes, *SOX9*, *COL2A1* and *ACAN,* and several catabolic genes including *ADAM-TS4&5,* and *INOS* were consistent with previous reports [40, 41]. However, we did not see changes in the expression of either *MMP13* or *PTGS2*, encoding *COX2.* Although not assayed here, it is known that traumatic impacts induce the increases in nitric oxide and PGE2, the product generated by COX2. We did not assay for NO or PGE2 directly, so it is possible the activity of COX2 is increased; however, the unchanged *MMP13* expression is confounding, despite using validated primers in the laboratory. Increases in *MMP13* expression are considered key hallmarks of osteoarthritic disease and cartilage injury. Traumatic impact has also been shown to increase *MMP13* expression in vitro and in vivo. The engineered cartilage constructs used in this study are simple both in matrix and in cell type. It is possible that cartilage contains a heterogenous cell population not represented by the MSC-derived population here and/or the cell–matrix interactions that contribute to increased *MMP13* expression after trauma are missing. We did, however, observe increases in signal by immunohistochemistry. Further studies are required to determine the mechanisms of *MMP13* gene induction, protein stability and activity as they relate to cell–matrix interactions in our chondrocyte constructs.

The comparison of the traumatic impact (as model of PTOA) to IL-1β is for comparative purposes, where IL-1β is a frequently employed model of osteoarthritic degeneration and sometimes as a model of OA [21, 42]. The engineered cartilage constructs used here responded as expected and as previously reported by us and others [43, 44]. That is, in comparison with unimpacted control samples, we observed an appropriate reduction in anabolic matrix products COL2 and ACAN and an increase in catabolic markers MM13 and ADAM-TS4 concomitant with increased inflammatory markers iNOS and CO2. These changes in gene expression as well as the reduction in construct GAG content compare with outcomes reported using explant cultures [27, 55].

The response to IL-1β differs in several ways. First, IL-1β is initially low, but progressively higher degradative state as reflected in the catabolic gene expression and histo/immunohistochemical data. Second, the induction of catabolic markers is much higher. The dose of IL-1β is high (10 ng/ml) and continuous, which may account for these differences with the traumatic load, which is a one-time, instantaneous insult. The fact that IL-1β induces MMP13 as expected suggests that this component of the physiological response to stress by the chondrocyte construct is present. Although MMP13 is considered a key investigative marker of OA, clinically its use for OA diagnosis and disease burden is not conclusive, as it overlaps with other conditions and protein profiles of control populations. In addition, the most common markers, such as collagen type-2 c-terminal neo-epitope CTXII generated by the activity of MMP13 on COL2, are only clearly diagnostic near the end-stage of the disease [45, 46]. Perhaps more prolonged, altered intracellular signaling or intra-tissue paracrine signaling is required to increase MMP13 gene expression in our system. Future evaluation of these hypotheses using the recently reported microJoint system will help elucidate these points [47–50].

The instantaneous nature of the impact also reveals a level of cell recovery not seen with continual IL-1β stimulation. The potential for a recovery phase was most clearly seen in the histological and immunohistochemical preparations, where pericellular GAG deposition seems to increase again on days 3 and 7, a phenomenon supported by the normalization of ACAN expression by day 7 after impact. Decreases in COL2 expression and increases in iNOS and PTGS2 also stopped by day 3 after traumatic load. The only biomarker that continued to show an increased “degenerative” arc was ADAM-TS4. This is in stark contrast to the progressive, highly patterns of common osteoarthritic biomarkers with continual IL-1β treatment. If these data hold true, the impact model may permit a more physiological evaluation of therapeutic interventions.

## Conclusions

In summary, we have shown that a traumatic load delivered to hydrogel-based engineered cartilage using adult human MSCs report many features of early degenerative changes in cartilage after trauma in vitro and in vivo. Such a model may prove important in the development of high-throughput testing systems for testing and optimizing DMOADs specifically for the treatment or prevention of PTOA, because it induces the early disease state in a mechanistically relevant manner.

## Supplementary Information

**Additional file 1:** **Figure** **S1**. A colony-forming unit assays of the MSC population used in this study. 30/100 of seeded cells generated colonies.

**Additional file 2:** **Figure** **S2**. (B) Tri-lineage differentiation of the MSC population used in this study. We assayed for: (A&D) chondrogenesis with Alcian Blue, (B&E) osteogenesis with Alizarin Red S, and (C&F) adipogenesis with Oil Red Staining (A-C magnification= 32x, D-F magnification=160x)

**Additional file 3:** **Figure** **S3**. Flow cytometric analysis of the MSC population used in this study. The MSC population was positive for (A) CD105, (B) CD73 and (C) CD90 and negative for (D) CD34, (E) CD45 and (F) CD31.

**Additional file 4:** **Figure** **S4**. Chondrogenic differentiation of engineered cartilage constructs. (A) Results of RT-PCR for 3 chondrogenic biomarker (COL2A1, SOX9 and ACAN) of day 0 and day 21 constructs. n=5/group. **, p < 0.01; *, p < 0.05 (B) H&E staining, Safranin O/ fast green staining and COL2 IHC for hydrogel constructs of day 0 and day 21.

## Data Availability

The datasets generated and analyzed during the current study are available from the corresponding author on reasonable request.

## Abbreviations

OA: Osteoarthritis
PTOA: Post-traumatic osteoarthritis
hBM-MSCs: Human bone marrow-derived mesenchymal stem cells
GelMA: Methacrylated gelatin
DMOADs: Disease-modifying osteoarthritis drugs
NSAIDs: Nonsteroidal anti-inflammatory drugs
MMPs: Matrix metalloproteinases
LAP: Lithium phenyl-2,4,6-trimethylbenzoylphosphinate
GM: Growth medium
DMEM: Dulbecco's Modified Eagle Medium
FBS: Fetal bovine serum
CM: Chondrogenic medium
MATE: Mechano-Active Tissue Engineering
GAGs: Glycosaminoglycans
MPS: Microphysiological systems
