# Supplementary figures and images for: Modeling early changes associated with cartilage trauma using human-cell-laden hydrogel cartilage models
Source: Stem Cell Res Ther. 2022 Aug 4;13:400. doi: 10.1186/s13287-022-03022-8 (PMC9351070; doi:10.1186/s13287-022-03022-8)

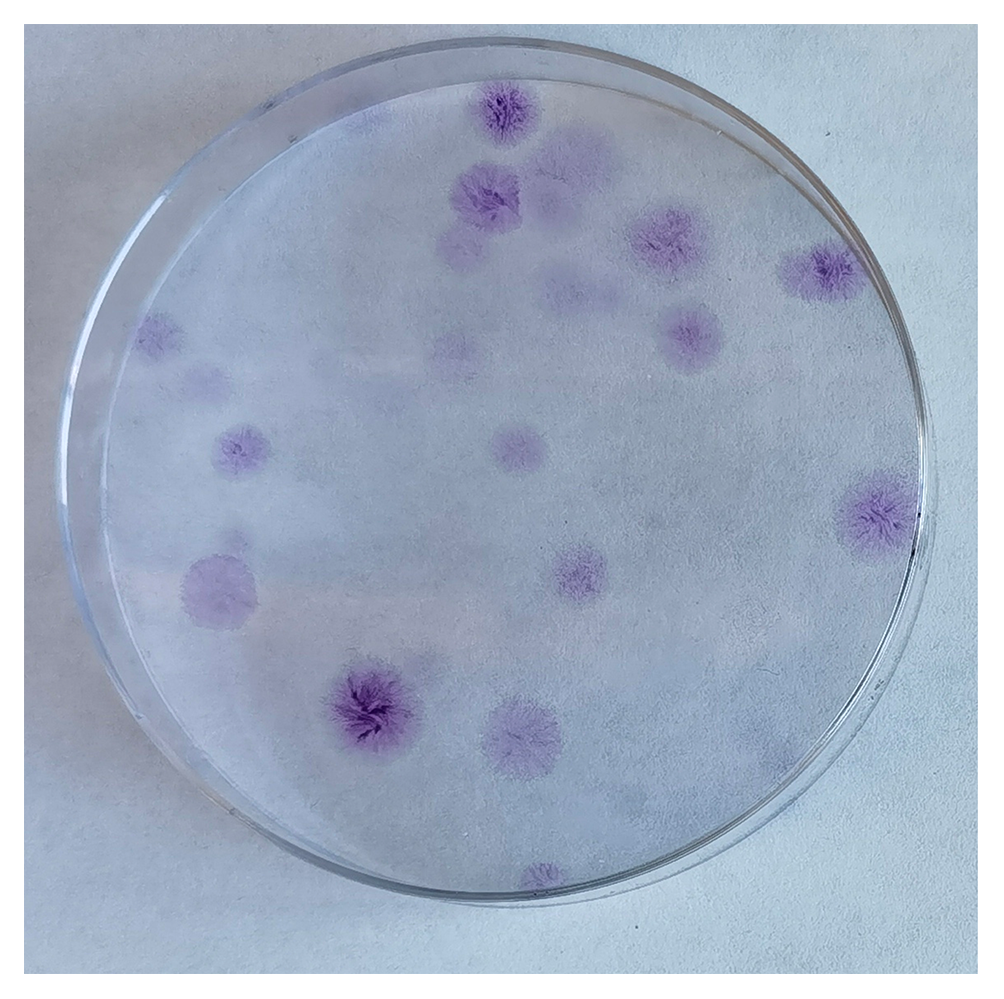

Supplement: Supplementary file 1 — Additional file 1: Figure S1. A colony-forming unit assays of the MSC population used in this study. 30/100 of seeded cells generated colonies. [file 13287_2022_3022_MOESM1_ESM.tif]

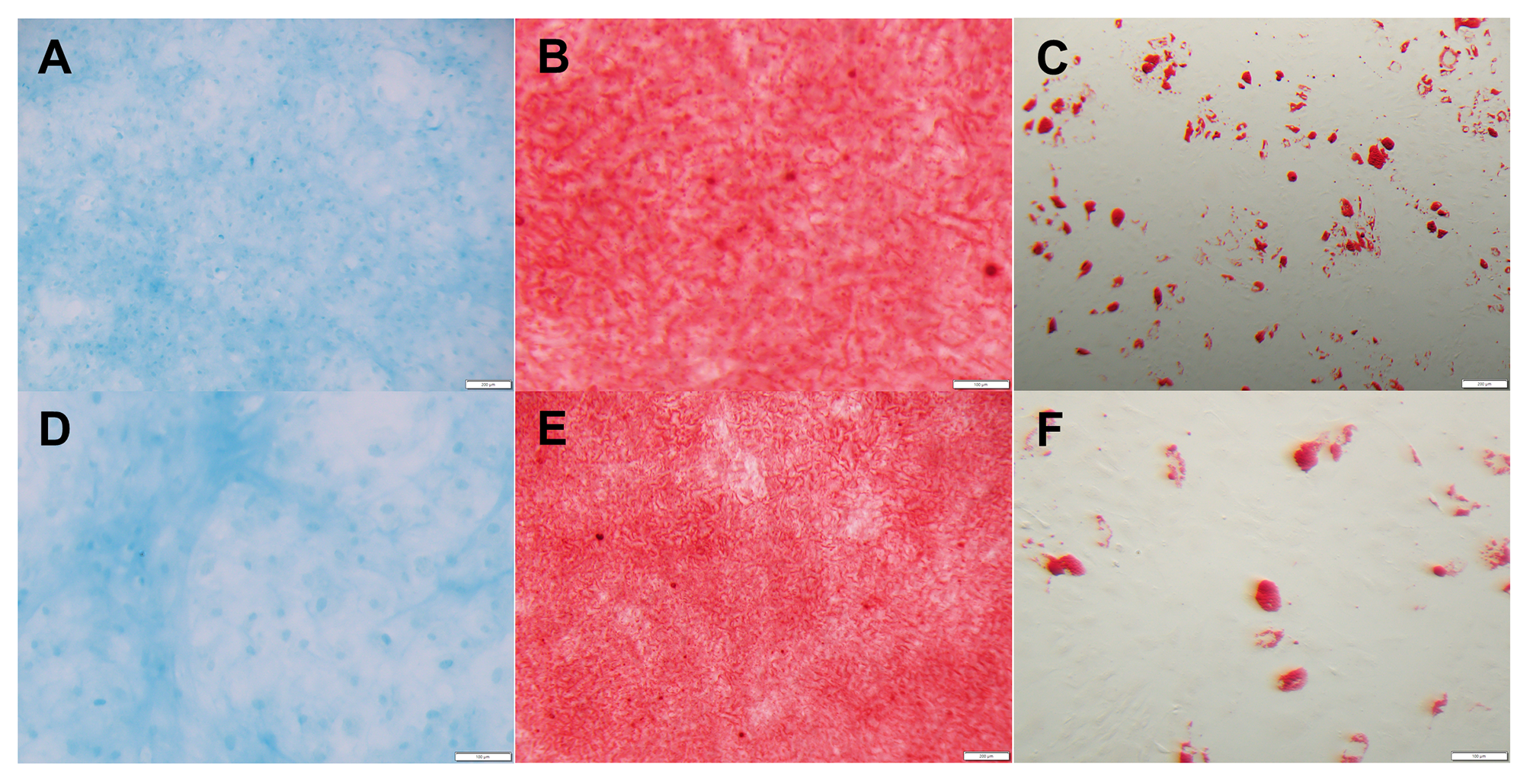

Supplement: Supplementary file 2 — Additional file 2: Figure S2. (B) Tri-lineage differentiation of the MSC population used in this study. We assayed for: (A&D) chondrogenesis with Alcian Blue, (B&E) osteogenesis with Alizarin Red S, and (C&F) adipogenesis with Oil Red Staining (A-C magnification= 32x, D-F magnification=160x) [file 13287_2022_3022_MOESM2_ESM.tif]

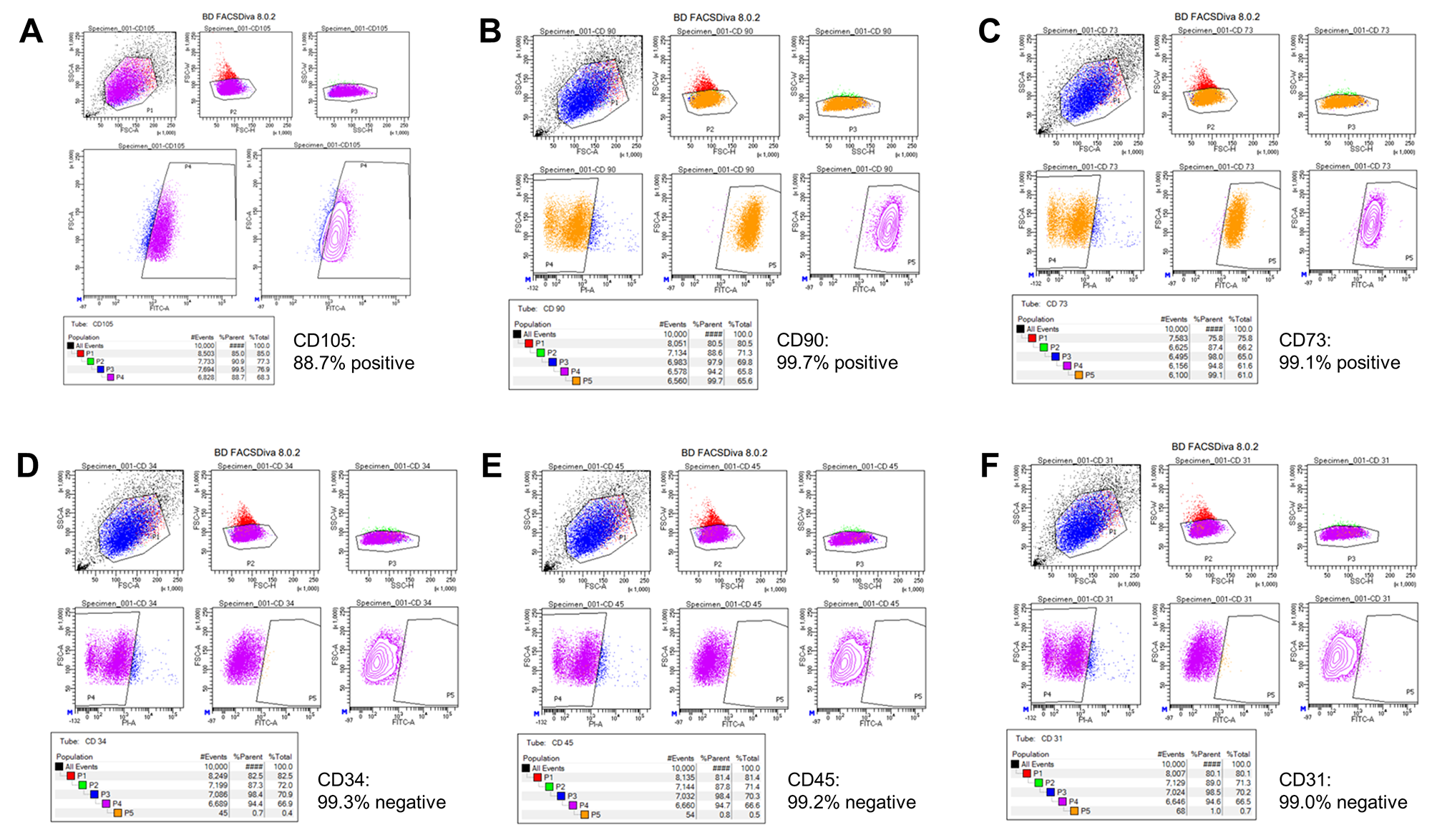

Supplement: Supplementary file 3 — Additional file 3: Figure S3. Flow cytometric analysis of the MSC population used in this study. The MSC population was positive for (A) CD105, (B) CD73 and (C) CD90 and negative for (D) CD34, (E) CD45 and (F) CD31. [file 13287_2022_3022_MOESM3_ESM.tif]

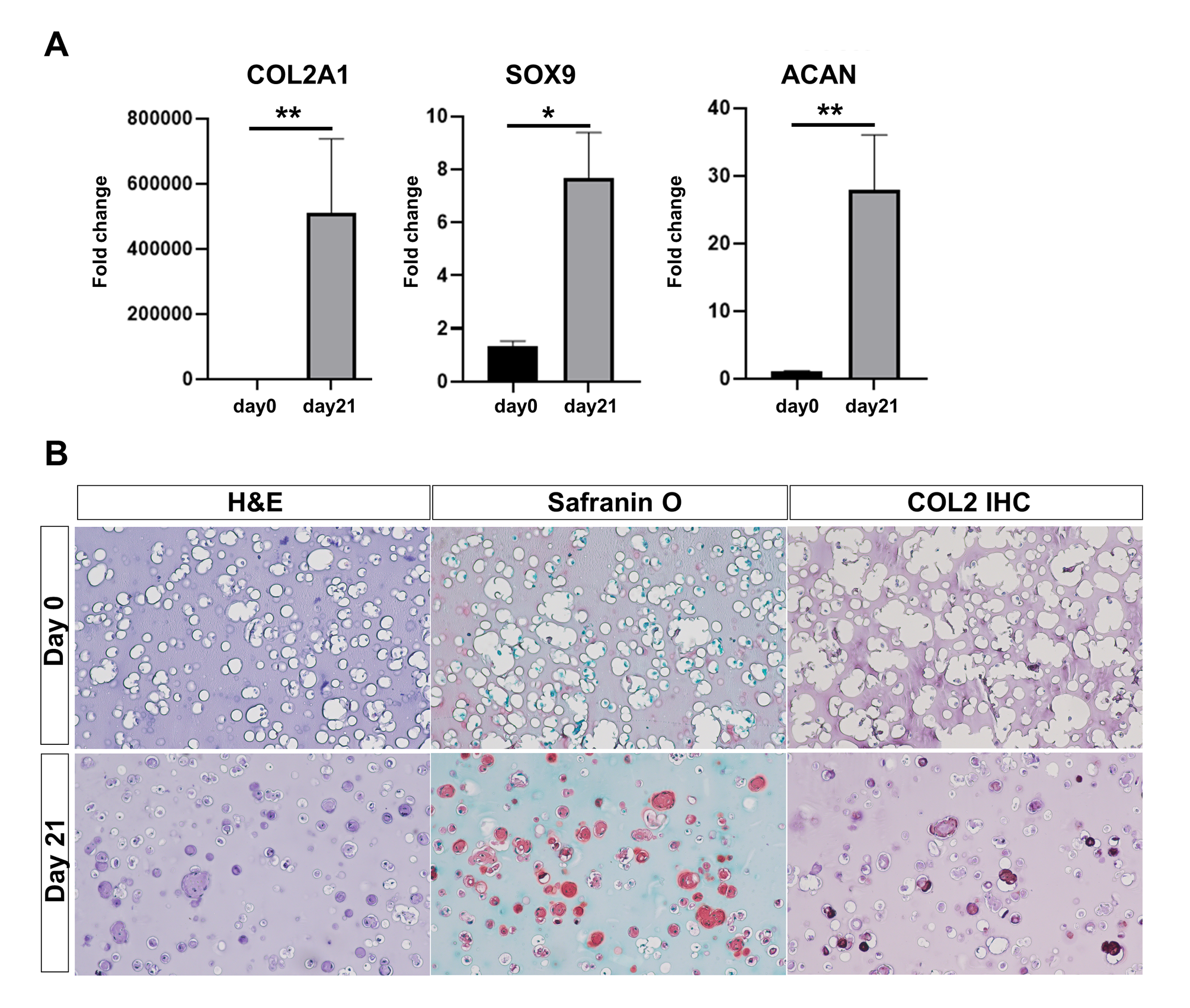

Supplement: Supplementary file 4 — Additional file 4: Figure S4. Chondrogenic differentiation of engineered cartilage constructs. (A) Results of RT-PCR for 3 chondrogenic biomarker (COL2A1, SOX9 and ACAN) of day 0 and day 21 constructs. n=5/group. **, p < 0.01; *, p < 0.05 (B) H&E staining, Safranin O/ fast green staining and COL2 IHC for hydrogel constructs of day 0 and day 21. [file 13287_2022_3022_MOESM4_ESM.tif]
